# Quest for malaria management using natural remedies

**DOI:** 10.3389/fphar.2024.1359890

**Published:** 2024-06-26

**Authors:** Qura Tul Ain, Nida Saleem, Nayla Munawar, Rukhsana Nawaz, Faiza Naseer, Sagheer Ahmed

**Affiliations:** ^1^ Shifa College of Pharmaceutical Sciences, Shifa Tameer-e-Millat University, Islamabad, Pakistan; ^2^ Shifa College of Medicine, Shifa Tameer-e-Millat University, Islamabad, Pakistan; ^3^ Department of Chemistry, College of Science, United Arab Emirates University, Al Ain, United Arab Emirates; ^4^ Department of Clinical Psychology, College of Medicine and Health Sciences, United Arab Emirates University, Al Ain, United Arab Emirates; ^5^ Department of Biosciences, Shifa Tameer-e-Millat University, Islamabad, Pakistan

**Keywords:** malaria, natural products, *Artemisia*, *Cinchona*, drug resistance

## Abstract

Malaria, transmitted through the bite of a *Plasmodium*-infected *Anopheles* mosquito, remains a significant global health concern. This review examines the complex life cycle of *Plasmodium*, emphasizing the role of humans and mosquitoes in its transmission and proliferation. Malarial parasites are transmitted as sporozoites to the human body by biting an infected female *Anopheles* mosquito. These sporozoites then invade liver cells, multiply, and release merozoites, which infect red blood cells, perpetuating the cycle. As this cycle continues, the affected person starts experiencing the clinical symptoms of the disease. The current treatments for malaria, including chloroquine, artemisinin-based combination therapy, and quinine, are discussed alongside the challenges of drug resistance and misdiagnosis. Although efforts have been made to develop a malarial vaccine, they have so far been unsuccessful. Additionally, the review explores the potential of medicinal plants as remedies for malaria, highlighting the efficacy of compounds derived from *Artemisia annua*, *Cinchona* species, and *Helianthus annuus* L., as well as exploration of plants and phytocompounds like cryptolepine, and isoliquiritigenin against drug-resistant *Plasmodium* species. Moreover, studies from Pakistan further highlight the diverse vegetal resources utilized in malaria treatment, emphasizing the need for further research into natural remedies. Despite the advantages of herbal medicines, including cost-effectiveness, and fewer side effects; their limitations must be taken into account, including variations in potency and potential drug interactions. The review concludes by advocating for a balanced approach to malaria treatment and prevention, emphasizing the importance of early detection, accurate diagnosis, and integrated efforts to combat the disease in the endemic regions.

## 1 Introduction

Malaria is one of the ancient diseases of humanity. Its historical records can be traced back as early as 2700 BC. By the early 1800s, malaria had become a global scourge, afflicting people across the world (Garcia, 2010). Despite scientific and medicinal advancements, malaria is still endemic in over 90 countries, and around 2 billion people are at risk of developing this infection annually (Finnigan, 2023). In the malaria hotspots, it is one of the leading causes of mortality, causing more than 1 million deaths worldwide (Pradines and Robert, 2019).

Malaria is a parasitic infection transmitted to humans by the *Anopheles* mosquito. When a mosquito bites a human, the parasite responsible for causing malaria is transmitted to the human body. *Plasmodium* exists in different forms, but only *Plasmodium falciparum*, *Plasmodium vivax*, *Plasmodium ovale*, and *Plasmodium malariae* are considered infectious. Among them, *P. falciparum* causes the most severe disease (Talapko et al., 2019). Malaria symptoms include headache, fever, myalgia, vomiting, and diarrhea. A severe infection can result in life-threatening conditions such as encephalopathy, high-grade fever, and gastroenteritis (Bartoloni and Zammarchi, 2012).

In endemic areas, malaria significantly impacts not only the health of individuals but also their socioeconomic profile. Due to poor living conditions and nutrition status, malaria disproportionately affects impoverished communities, exacerbating the challenges faced by vulnerable social groups (Suh, Kain, and Keystone, 2004). Malaria stands as one of humanity’s most fatal afflictions. The horrors unleashed by this ancient disease persist to the present day. Unfortunately, an effective and potent cure for the infection continues to elude us. The challenges span from complexities of early detection of malaria to obstacles to formulating a suitable remedial strategy, collectively presenting a formidable hurdle in its treatment.

Quinine was the first drug for malaria treatment (Weinke et al., 1992). However, with advancements in medicine, treatment options have also evolved. Currently, chloroquine, artemisinin, proguanil, primaquine, mefloquine, and sulfonamides are used to manage malaria (Gilles and Warrell, 1996). However, there is still no empirical drug for it, as these drugs are plagued by resistance and undesirable side effects (Castelli, Tomasoni, and Matteelli, 2012).

Furthermore, homology modeling and molecular dynamics simulations provide valuable insights into *P. falciparum* chloroquine resistance transporter (*PfCRTs*) and its interactions with chloroquine (Patel and Roy, 2021). *In vitro* assays reveal increased IC_50_ (a measure of potency) values for mefloquine, suggesting diminished parasite susceptibility. Moreover, the concurrent emergence of resistance to artesunate, coupled with mefloquine resistance, underscores the urgent requirement for alternative approaches to malaria treatment (Rogers et al., 2009b).

For centuries, natural products have been utilized in the treatment of various conditions. This is particularly true for malaria because many anti-malaria drugs are derived from plants, including quinine and artemisinin, which are crucial in malaria control (Kingston and Cassera, 2022). However, the reduced effectiveness of the old therapies due to various reasons including the emergence of resistance in the parasite necessitates the search for newer agents to treat malaria. This article aims to review natural remedies that can be used to treat malaria including the resistant strains while emphasizing the challenges faced by the empirical class of drugs.

## 2 Epidemiology

**FIGURE 1:**
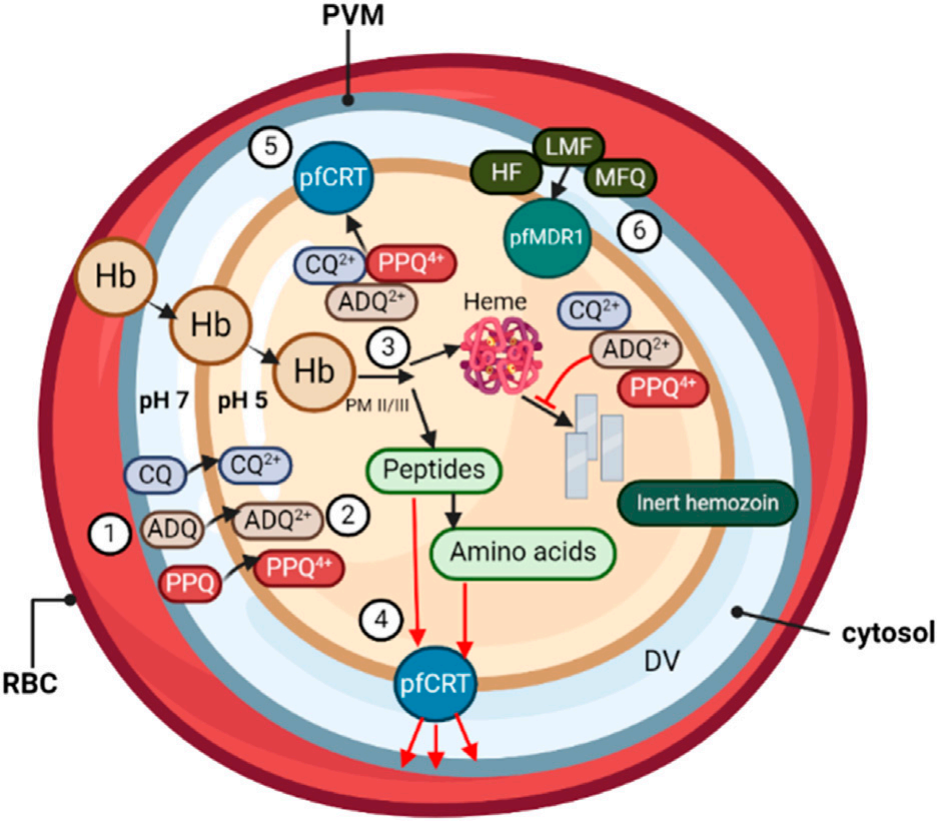
During its asexual blood stage, the *P. falciparum* parasite, surrounded by its parasitophorous vacuole membrane (PVM), develops within the host RBCs. (❶) Quinoline-based antimalarials, including chloroquine (CQ), amodiaquine (ADQ), and piperaquine (PPQ), concentrate from the parasitic cytosol (pH ∼7) into the digestive vacuole (DV) (pH ∼5.2). (❷) Once inside the DV, weak-base drugs are protonated, unable to passively diffuse out through the DV membrane. (❸) Protonated drug molecules bind to toxic heme by-products and grooves on hemozoin crystals in the DV, resulting in >1,000-fold drug accumulation due to pH trapping and heme-binding. (❹) The DV membrane protein *PfCRT* is believed to be involved in transporting peptides released from hemoglobin digestion into the parasite cytosol. (❺) In drug-resistant parasites, mutations in *PfCRT* enable the efflux of protonated drug molecules out of the DV, away from their heme target. (❻) DV membrane transporter *PfMDR1* mutations influence parasite susceptibility by redirecting drugs such as halofantrine (HF), lumefantrine (LMF), and mefloquine (MFQ) into the DV, away from their primary site of action.

**FIGURE 2:**
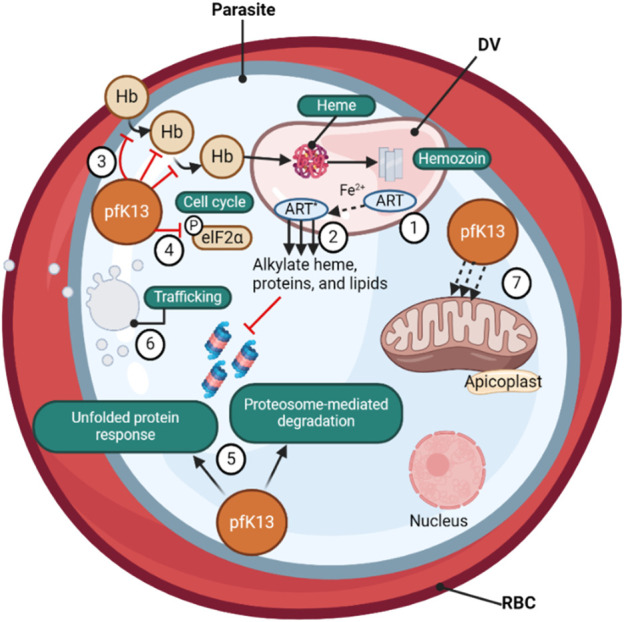
(❶) *Artemisinin*-based (ART) drugs are activated by cleavage of their endoperoxide by iron protoporphyrin IX (Fe^2+^-heme), a product of parasite-digested hemoglobin. (❷) The Fe^2+^-heme- ART carbon-centered radicals alkylate and damage a multitude of parasite proteins, heme, and lipids and inhibit proteasome-mediated protein degradation. *PfK13* mutations, located primarily in the β-propeller kelch domain, confer ART resistance. (❸) The loss of *PfK13* function provided by mutations has been shown to cause reduced endocytosis of host Hb and (❹) to extend the duration of ring-stage development, perhaps via PK4-mediated eIF2α phosphorylation, resulting in lowered levels of Hb catabolism and availability of Fe^2+^-heme as the drug activator, reducing ART activation (❺) *PfK13* mutations activate the unfolded protein response, maintain proteasome-mediated degradation of polyubiquitinated proteins in the presence of ART, (❻) remove drugs and damaged proteins through an increase in PI3K-mediated vesicular trafficking. (❼) *PfK13* may also help regulate mitochondrial physiology and maintain membrane potential during drug-induced ring-stage quiescence. The asterisk signifies the activated form of ART (Wicht, Mok, and Fidock, 2020).

According to the World Health Organization’s annual report on malaria, there were more than 240 million malaria cases worldwide. From 2000 to 2021, around 2 billion people were estimated to have suffered from the infection. However, The number of total malaria cases globally increased in 2021, from 245 million in 2020 to 247 million in 2021 (WHO, 2023a; 2022). Most cases are concentrated in the African region (95%), with around 2% in the Southeast Asian region. Approximately 29 countries accounted for 96% of malarial deaths (Figures 1,2; Table 1).

**TABLE 1:** Malaria cases and deaths by geographical region (WHO Annual Report, 2022).

Region	Cases (%)	Deaths (%)
Africa	95	96
Southeast Asia	2	1
Eastern Mediterranean Region	1	≤1
Others	1	≤1

A significant burden of malaria cases is shared by four countries, namely, Nigeria (27%), the Democratic Republic of Congo (12%), Uganda (5%), and Mozambique (4%) (Commission, 2023). Similarly, around 96% of deaths due to malaria occurred in four countries, i.e., Nigeria, the Democratic Republic of Congo, Niger, and the United Republic of Tanzania (WHO, 2023a). According to the WHO, more than 94% of malaria cases (233 million) and 95% of deaths occurred in the African region (Organization, 2023).

However, the epidemiology of malaria in India is distinct due to several features of the *Plasmodium* parasites, *Anopheles* vectors, ecoepidemiological aspects conducive to disease transmission, and susceptible humans living in rural and forested areas (Khan MuhammadImran et al, 2023). Notably, in 2021, India accounted for 79% of malaria cases and about 82.4% of all malaria deaths in the South-East Asia Region (Organization, 2022).

Lastly, a systematic review for finding out the prevalence of malaria (from 2001 to 2021) estimated pooled malaria prevalence in Pakistan to be 23.3%, with *P. vivax*, *P. falciparum*, and mixed infection rates of 79.13%, 16.29%, and 3.98%, respectively. Moreover, the analysis marked Karachi as the hotspot for malaria with a prevalence rate of 99.79% (Khan Nikhat et al., 2023). This data suggests that malaria is still endemic in most parts of the world. Although the infection rates have decreased over the years, it remains one of the most challenging health problems faced by the global south.

## 3 Pathophysiology of malaria

**FIGURE 3:**
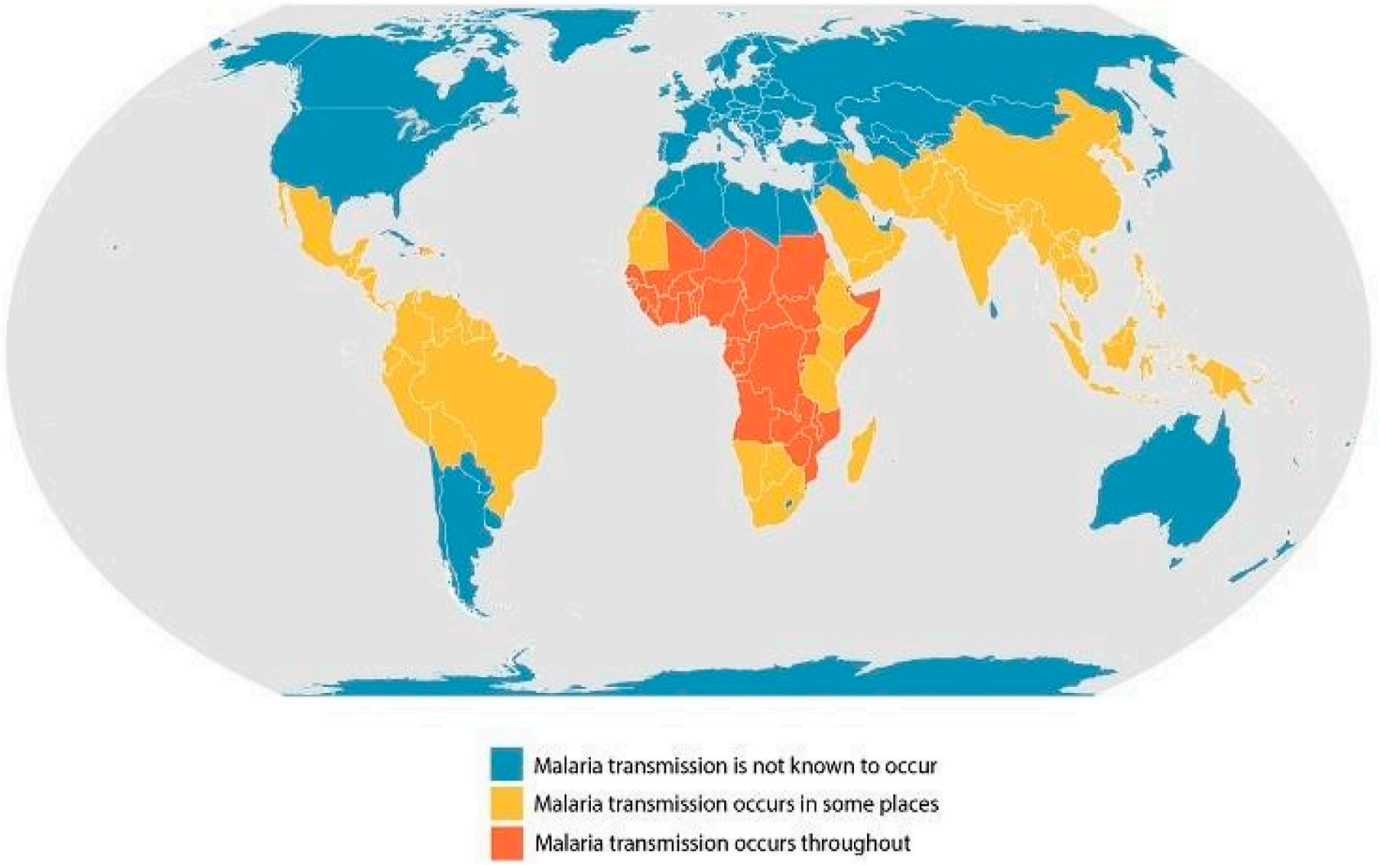
Geographical distribution of malaria (Centre for Disease Control and Prevention).

The life cycle of *Plasmodium* involves humans and mosquitoes, where the human body acts as a site for asexual reproduction, while mosquitoes are involved in the sexual stage of their lifecycle (Figure 3). Mosquitoes serve as vectors and transmit *Plasmodium* to humans, where it causes malaria (Giribaldi et al., 2015). *Plasmodium* is transmitted to humans by the female *Anopheles* mosquito. There are more than 450 species of *Anopheles*, but about 68 are involved in the transmission of the disease (Giribaldi et al., 2015).

Once it bites a human, the sporozoite form of the parasite is transmitted to the host.

The sporozoite form circulates via the bloodstream and resides in the liver, leading to malaria infection (Menard, 2005). The sporozoites undergo asexual reproduction, resulting in merozoites. This stage is categorized as the “liver stage” with no clinical symptoms. In the case of *P. falciparum* and *P. malariae*, the merozoites are released into the bloodstream, leading to the erythrogenic stage, where malaria infection symptoms start appearing. In the case of *P. vivax* and *P. ovale*, the parasite may enter a dormant hypnozoite stage, significantly increasing the risk of reinfection (Markus, 2011). As the merozoites invade RBCs, they rapidly grow and develop into ring-like forms called “trophozoites” that can be identified via Giemsa staining (Giribaldi et al., 2015).

## 4 Current treatments

**FIGURE 4:**
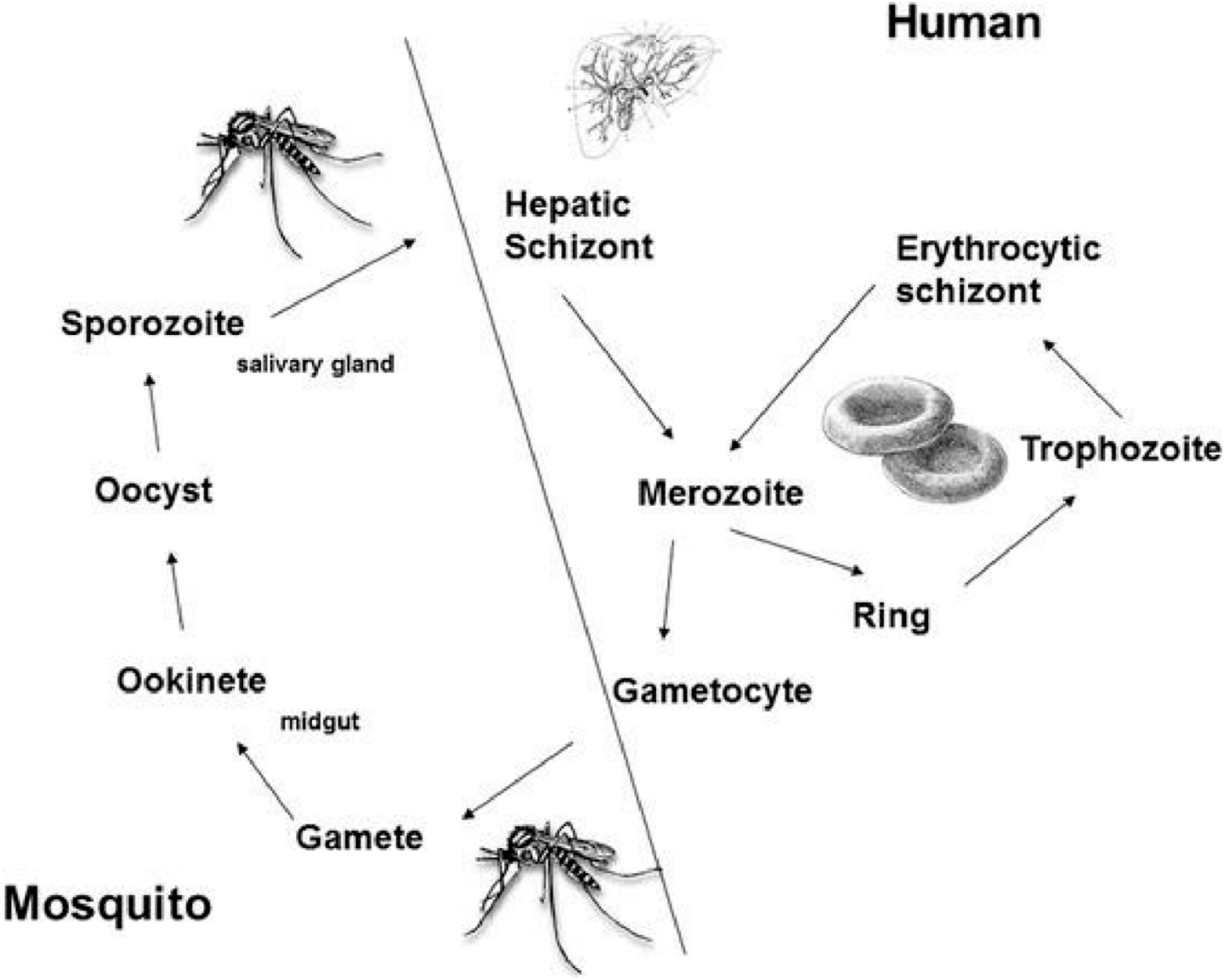
The life cycle of *Plasmodium* in humans and *Anopheles* mosquito.

The treatment and management of malaria depend upon accurate and timely diagnosis since its symptoms can differ significantly from one person to another (Bartoloni and Zammarchi, 2012). The clinical objectives of treating uncomplicated malaria are to cure the infection as rapidly as possible and to prevent progression to severe disease. “Cure” is defined as the elimination of all parasites from the body (WHO, 2023b). Current guidelines recommend that non-*falciparum* malaria should be treated with a 3-day course of oral chloroquine and oral analgesics (Chiodini et al., 2007). Primaquine is considered the most effective drug to eliminate *P. vivax* and *P. ovale*, even for the dormant hypnozoites in the liver (Wångdahl et al., 2022). Artemisinin-based combination therapy is used to treat uncomplicated *P. falciparum* malaria infection and the artemisinin-lumefantrine combination is a drug of choice for its treatment (McIntosh et al., 1996). Severe *falciparum* infection is treated with intravenous (IV) drugs until the patient can be switched to oral drugs. The treatment for severe malaria in adults and children is IV artesunate (Trampuz et al., 2003). The chemical structures of some of these drugs are provided in Figure 4.

Furthermore, treatment options for pregnant women and children afflicted with malaria are different and should be used with caution to avoid undesirable side effects (Whitty, Edmonds, and Mutabingwa, 2005). In addition, severe malaria infections in pediatrics should be treated with empirical broad-spectrum antibiotics. In case of uncomplicated infection, a combination of artemether-lumefantrine can be utilized as the first line of treatment (Chiodini et al., 2007). In the first trimester of pregnancy, uncomplicated malarial infection should be treated with quinine-clindamycin combination therapy. In the second and third trimesters of pregnancy, artemether-lumefantrine treats uncomplicated *P. falciparum* malaria (Whitty, Edmonds, and Mutabingwa, 2005). For severe malaria, in any trimester of pregnancy, artesunate is usually preferred over quinine. Severe malarial infection by *P. falciparum* in pregnancy can potentially result in stillbirth or early delivery, so it's advisable to start the treatment as early as possible, under the supervision of a specialist doctor (Nunes and Scherf, 2007).

Furthermore, in patients co-infected with HIV/AIDS and uncomplicated *P. falciparum* malaria, artesunate + sulfadoxine-pyrimethamine is not recommended if they are being treated with co-trimoxazole. Additionally, artesunate + amodiaquine is not recommended if they are being treated with efavirenz or zidovudine (WHO, 2023a; Obeagu et al., 2024). Another critical treatment case is malaria-tuberculosis co-infection. Severe drug interaction, for example, rifampicin-induced liver injury may be worsened by amodiaquine-containing artemisinin-based combination therapy, complicating the treatment plan (Adedeji et al., 2018). Currently, there is insufficient evidence to modify the existing dosing recommendations based on mg/kg body weight. However, due to the higher risk of recrudescent malaria infections in these patients, close monitoring is advised (World Health, 2023).

Moreover, the November 2022 update to treatment recommendations for malaria includes several key points. Firstly, it suggests the use of artesunate-pyronaridine as an artemisinin-based combination therapy option for treating uncomplicated *P. falciparum* malaria, despite low-certainty evidence. Additionally, pregnant women diagnosed with uncomplicated *P. falciparum* malaria during the first trimester are advised to undergo treatment with artemether-lumefantrine, again supported by a strong recommendation despite low certainty evidence. Additionally, to prevent relapse in cases of *P. vivax* or *P. ovale* malaria in children and adults (with specific exceptions), primaquine at a dosage of 0.5 mg/kg/day for 7 days was strongly recommended. Conversely, the update advises against using primaquine at a dosage of 1.0 mg/kg/day for 7 days to prevent relapse in *P. vivax* or *P. ovale* malaria, based on a conditional recommendation and very low-certainty evidence (Organization, 2023).

In addition to antipyretics, like acetaminophen, and anti-emetics, anti-seizure medications are also advised. Importantly, generalized seizures are more common in children with *P. falciparum* malaria than in those with malaria caused by other species. Usually, parenteral or rectal benzodiazepines or intramuscular paraldehyde are given. However, prophylactic use of anti-convulsants has no firm empirical foundation in otherwise uncomplicated malaria and therefore is not recommended (World Health, 2023).

## 5 Challenges associated with current treatments

One of the main challenges faced by clinicians in malaria treatment is drug resistance developed by *Plasmodium*. *P. falciparum*, responsible for severe malarial infection, has developed resistance to many drugs, including chloroquine, mefloquine, and quinine (Vestergaard and Ringwald, 2007). Chloroquine is considered the most cost-effective drug for malaria management, but recent data suggests that the treatment failure rate with chloroquine is more than 70% due to rapid drug-resistance development (Vestergaard and Ringwald, 2007). Amodiaquine, an essential part of the drug combinations used for treating malaria, is also facing the same threat.

Moreover, malaria misdiagnosis is another major challenge in combatting the epidemic. Accurate use of diagnostic procedures may not only lead to early infection treatment but also help select the correct antimalarial drug regimen against a particular pathogen (Shahbodaghi and Rathjen, 2022).

Vector control is one of the bigger challenges in the fight against malaria. In addition to outdoor transmission, growing levels of insecticide resistance in targeted vectors threaten the efficacy of long-lasting insecticidal nets and indoor residual spraying. These methods reduce malaria incidence but generally have little impact on malaria prevalence. Larvicidal treatments can be useful but are not recommended for rural areas. The research needed to improve the quality and delivery of mosquito vector control (Benelli and Beier, 2017). Due to the drug-drug interaction of antimalarial drugs, caution is always needed before prescribing. When treating malaria in someone who is also taking anti-tuberculosis medications, one must be careful to prevent harmful drug interactions. This is because the anti-tuberculosis drugs, such as rifampicin and isoniazid, can cause liver damage, and this risk could be heightened by using artemisinin-based combination therapy with amodiaquine to treat malaria, potentially leading to more severe outcomes (Adedeji et al., 2018).

## 6 Mechanisms of resistance

There is a plethora of factors behind anti-malaria drug resistance. The mechanisms of resistance to chloroquine in malaria parasites often involve mutations in the *PfCRT* gene, which reduce the drug accumulation within the parasite, diminishing its efficacy and facilitating parasite survival (Patel and Roy, 2021). In mosquitoes, exposure to chloroquine can lead to alterations in innate immunity, potentially affecting their ability to treat *Plasmodium* infections. Moreover, chloroquine disrupts normal apoptotic processes in mosquito midgut cells, potentially facilitating parasite survival and development within the mosquito host. These changes in parasite susceptibility and mosquito physiology can significantly influence malaria transmission dynamics and the proliferation of drug-resistant strains (Abrantes et al., 2008). The resistance mechanisms to mefloquine in *P. falciparum* involve mutations in the *pfmdr1* gene, impacting drug transport and efficacy. Amplification of *pfmdr1* correlates with heightened mefloquine resistance and treatment failure rates. Prolonged parasite clearance post-mefloquine treatment signifies potential resistance development. *In vitro* assays indicated elevated IC_50_ values for mefloquine, indicative of decreased parasite susceptibility. Furthermore, emerging resistance to artesunate, alongside mefloquine resistance, highlights the pressing need for alternative malaria treatment approaches (Rogers et al., 2009a).

Studies regarding molecular mechanisms involved in anti-malaria drug resistance found the involvement of variation in *P. vivax* multidrug resistance gene 1 (*PvMDR1*) (Reed et al., 2000; Chaorattanakawee et al., 2017; Li et al., 2020), *P. vivax* chloroquine resistance transporter (*PvCRT*) (Sá et al., 2019), *P. vivax* dihydrofolate reductase-thymidylate synthase (*PvDHFR*-*TS*), dihydropteroate synthase (*PvDHPS*) (Hawkins et al., 2007; Auburn et al., 2018; Auburn et al., 2019), and *P. vivax* multidrug resistance protein 1 (*PvMRP1*) (Bright et al., 2013; Flannery et al., 2015) in mediating drug resistance (Buyon, Elsworth, and Duraisingh, 2021). Furthermore, chemoprevention or chemoprophylaxis strategies may also inadvertently exacerbate the resistance problem. Malaria chemoprevention interventions do not always lead to favorable outcomes; counter-intuitively, they may increase the prevalence of resistance-associated genetic mutations (Plowe, 2022).

Drugs that are effective in adults cannot be used to treat malaria in pregnancy and children. This is another area of concern as many times, the same drugs are prescribed in all age groups leading to increased risk of adverse reactions and potentially life-threatening consequences during pregnancy (Phillips-Howard and Wood, 1996). Moreover (Acosta et al., 2020), assessed the relationship among drug dose, timing, and drug resistance in the malaria model. The analysis revealed that the higher dose administration may lead to high-level resistance. In addition, altering treatment timing can also influence the risk of resistance emergence. The study concluded that identifying the “right” time and dose was crucial for maximizing clinical goals (Acosta et al., 2020). In addition, an investigation focusing on the correlation between anti-malarial market characteristics and the emergence of arteminisin revealed that there was an urgent need for tight regulation to delay the emergence and spread of arteminisin (Guissou et al., 2023).

## 7 A glance at malaria vaccine-related advancements and development

About half of the world’s population is still at risk of contracting malaria, so making an effective malaria vaccine is the need of the hour, a goal that remains elusive for now. In 2021 the WHO approved the first malaria vaccine, RTS,S/AS01 vaccine (Mosquirix™), for widespread use. However, development continues on promising candidates such as R21, PfSPZ, and *P. vivax* vaccines (El-Moamly and El-Sweify, 2023).

R21/Matrix-M™ Malaria Vaccine is a product of collaboration between the University of Oxford and Novavax. By reaching 77% effectiveness in Phase 2 clinical trials, R21 demonstrated remarkable efficacy. Moreover, it is designed to be low-dose, accessible, and cost-effective, for use in children under 3 years of age (Aderinto et al., 2024). In addition to these developments, many researchers have emphasized the need for developing asexual blood-stage malaria vaccine development against *Plasmodium* (Takashima et al., 2024).

Thus far, two types of vaccines have been developed. One type, called subunit vaccines, like RTS,S, and R21, target a specific part of the parasite, but they have not been very effective at stimulating the immune system to protect against malaria for a long time (MacMillen et al., 2024). Another type of vaccine called whole-organism vaccines, such as Radiation Attenuated Sporozoites (RAS) exhibited better protection, but they are difficult and expensive to make since they need to be grown inside mosquitoes. Moreover, these vaccines require a series of IV doses administered over multiple clinic visits (Sissoko et al., 2022). To overcome this, researchers proposed a novel strategy called a “prime-and-trap” approach, involving using two different vaccines together. *In vivo*, the first vaccine (the priming dose) a single dose of a self-replicating RNA encoding full-length *P. yoelii* CS protein was delivered using advanced Nano carriers called LION™. The second vaccine (the trapping dose) consists of an attenuated malaria parasite, delivered as a whole-organism vaccine (WO RAS). Interestingly, an accelerated regimen, i.e., either 5-day or same-day immunization induced a strong immune response. Notably, mice vaccinated on the same day experienced a 2-day delay in the appearance of parasites in their blood and achieved 90% protection against a 3-week spz challenge. Moreover, this same-day regimen provided 70% protection against the 2-month spz challenge. This innovative approach may aid in developing vaccines that require fewer doses and provide long-lasting protection against malaria (MacMillen et al., 2024).

Furthermore, malaria transmission-blocking vaccines, which interrupt malaria transmission from one person to another, have also been tested in malaria-endemic areas of Africa. The study found that all the tested formulations were safe and well tolerated in healthy adults. Particularly, ProC6C-AlOH/Matrix-M vaccine elicited the highest levels of functional antibodies, requiring further investigation (Tiono et al., 2024).

## 8 Medicinal plants as remedies for malaria

**FIGURE 5:**
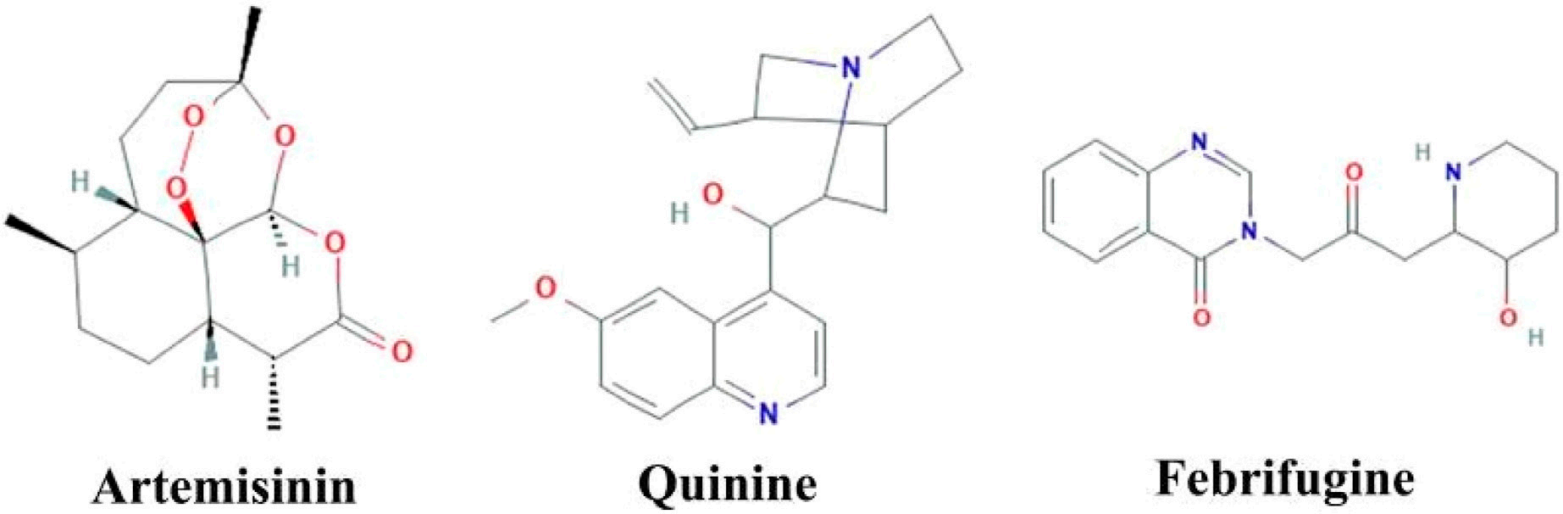
Structure of 3 common plant-derived anti-malaria drugs.

Herbal medicines have many proven advantages over synthetic drugs, as the former is more cost-effective, has fewer side effects, and is more efficient (Mohammadi et al., 2020). Vegetal species have been used for centuries for different therapeutic purposes. Researchers from all over the world have identified numerous plant-based products that are effective in managing various diseases including malaria (Lemma et al., 2017; Hassan et al., 2021; Afzal et al., 2022; Nawaz, Ishtiaq, and Anwar, 2022). Derived from the plant named *Artemisia annua*, artemisinin has revolutionized the treatment of malaria (Phillipson, 1990). Similarly, various herbal medicines are derived from *Cinchona* species, with quinine being one of them (White, 1985). Febrifugine, another antimalarial drug, is derived from the plant *Dichroea febrifuga* Lour. (Farnsworth and Soejarto, 1991). Artemisinin and quinine contain bioactive compounds/phytochemicals, alkaloids and terpenoids that are effective for the treatment of malaria infection (Moyo et al., 2020). The structural formula for artemisinin, quinine, and febrifugine is shown in Figure 5. Here we describe some of the most important plants with promising anti-malarial activities.

### 8.1 *Cryptolepis sanguinolenta* (Lindl.) Schltr.

*Cryptolepis sanguinolenta* (Apocynaceae) leaves are used in the treatment of malaria. In 1996, cryptolepine was first isolated from the roots of *C. sanguinolenta*, and it inhibits DNA synthesis, which is the main reason for its antimalarial action (Osafo, Mensah, and Yeboah, 2017). In a study, both cryptolepine and isocryptolepine exhibited effective inhibition of *P. falciparum in vitro*, regardless of the strain’s resistance to chloroquine. Cryptolepine demonstrated slightly better performance in killing the parasite, with an IC_50_ ranging from 0.2 to 0.6 μM, compared to isocryptolepine’s (another alkaloid from *C. sanguinolenta*) IC_50_ of about 0.8 μM. The antimalarial activity of cryptolepine was also confirmed *in-vivo* rodent malarial parasites. The results showed that cryptolepine was effective against even the resistant varieties of *Plasmodium* parasites (Grellier et al., 1996; Table 3). This plant has been used in traditional African medicine to manage malaria, showing promising results (Mohammadi et al., 2020).

### 8.2 *Artemisia annua* L.

In a clinical study, patients with severe malaria who responded to neither Artemisinin combination Therapy (ACT) nor IV artesunate were treated with dried leaves of *A. annua* (Asteraceae). At the dose of 0.5g, twice daily for 5 days, the *A. annua* dried-leaf oral solution. Another study investigated *A. annua* anti-malaria potentials by subjecting its aqueous and hydroalcoholic extracts to *in vitro* and *in vivo* evaluations. Interestingly, the hydroalcoholic extract was found to be more active (IC_50_ = 3.27 ± 1.42 nM) than artemisinin (IC_50_ = 5.10 ± 1.89 nM) *in vitro*. However, the *in vivo* investigations using *Plasmodium berghei* NK 173 infected mice revealed that the aqueous extract of *A. annua*, with artemisinin content of 20 mg/kg, demonstrated comparable efficacy to pure artemisinin administered at a dosage of 140 mg/kg (Zime-Diawara et al., 2015). A series of clinical trials have proven the efficacy of these compounds, making these drugs one of the preferred choices of clinicians for eradicating *Plasmodium* parasites from the body (Chiodini et al., 2007). Information about several drugs derived from *Artemisia* species is provided in Table 2.

**TABLE 2:** Important commercial drugs derived from *Artemisinin*.

*Artemisinin* commerical derivatives	Brand name	Manufacturer	Pharmaceutical dosage form
Artemether	Artenam	Arenco	Ampoule
	Artem	Hilton	Ampoule
Artemether plus lumefanterine	Coartem	Novartis	Tablet
	Lumartem	Cipla	Tablet
Artesunate	Asunate	Adley	Vial for injection
	Falcigo	Zindus cadila	Vial for injection
Artesunate plus amodiaquine	Co-artesun	Guilin	Tablet
Dihydroartemisinin	Alaxin	GVS labs	Tablet
Artesunate plus mefloquine	Artequin	Acino Switzerland	Tablet

A study was conducted by (Elfawal et al., 2015) to demonstrate the efficacy of the whole *A. annua* plant, as a malaria therapy, is more potent than a comparable dose of pure artemisinin in a rodent malaria model. For this purpose, in the rodent malaria model, *P. chabaudi* was chosen due to its susceptibility to both whole-plant *A. annua* and artemisinin treatment. *P. yoelii* was selected as an artemisinin-resistant strain for challenge experiments. The study design consisted of five animal groups, 4 serving as treatment groups and 1 as control. Treatments included dried *A. annua* plant, and pure artemisinin, administered orally. The experiment utilized two doses of pure artemisinin and two doses of whole plant. The low dose of pure artemisinin was 24 mg/kg, while the high dose was 120 mg/kg. Similarly, the low dose of whole-plant *A. annua* corresponded to 24 mg of artemisinin per kilogram of body weight, and the high dose was 120 mg/kg. The results revealed that a stable resistance to the whole plant was achieved; three times slower than stable resistance to artemisinin. *A. annua* treatment was even more resilient than the double dose of artemisinin. This resilience was attributed to the potential evolutionary refinement of the plant’s secondary metabolic products into a redundant, multi-component defense system (Elfawal et al., 2015; Table 3).

**TABLE 3:** Plants against resistant *Plasmodium* species.

Scientific name and family	Common name	Part used and extract	Dosing	Study type	Citation
*Cryptolepis sanguinolenta* (Apocynaceae)	Nibima, Yellow-dye root	**Part:** Root **Extract:** Aqueous	Cryptolepine IC_50_: 0.2–0.6 µM Isocryptolepine IC_50_: 0.8 µM	*In vitro*	Grellier et al. (1996)
*Artemisia annua* (Asteraceae)	Sweet wormwood	**Part:** Whole plant **Extract:** Aqueous	24mg/kg-120 mg/kg	*In vivo*	Elfawal et al. (2015)
*Cyperus articulates* (Cyperaceae)	Jointed flatsedge	Volatile oil	IC_50_ against W2 (resistant strain): 1.21 μg/mL	*In vitro*	Silva et al. (2019)
*Pogostemon cablin* (Lamiaceae)	Patchouli	**Part:** Dried Ariel parts **Extract:** Ethanol	IC_50_ against K1 (resistant) 24.49 μg/mL	*In vitro*	Phuwajaroanpong et al. (2020)
• *Toddalia asiatica* (Rutaceae) •*Rhamnus prinoides* (Rhamnaceae) •*Vernonia lasiopus* (Compositae)	• Orange Climber •Gesho •-	**Part:** • Root bark • Leaves • Root bark **Extract:** Aqueous	Dose: 500 mg/kg	*In vivo*	Muregi et al. (2007)
• *Maytenus senegalensis* (Celastraceae) •*Rhamnus staddo* (Rhamnaceae)	• Spike thorn •Staddo	**Part:** • Root bark • Leaves and root bark **Extract:** Aqueous	Dose: 500 mg/kg	*In vivo*	Muregi et al. (2007)
• *Citrullus colocynthis* (Cucurbitaceae) •*Physalis alkekengi* (Solanaceae) •*Solanum nigrum* (Solanaceae)	• Bitter apple •Chinese lantern •Black nightshade	**Part:** • Fruits • Leaves and fruit • Fruits **Extract:** Methanolic	IC_50_ • 6.9 μg/mL • 18.67 μg/mL • 13.08 μg/mL	*In vitro*	Haddad et al. (2017)
*Glycyrrhiza glabra* (Fabaceae)	Licorice	**Part:** Root **Extract:** Ethyl acetate and chloroform	Isoliquiritigenine IC_50_ 7.2 μg/mL Isoliquiritigenine + Chloroquine IC_50_ 1.90 μg/mL Isoliquiritigenine + Artemisinin IC_50_ 1.75 ng/mL	*In vitro*	Kumar et al. (2024)
*Cuscuta reflexa* (Convolvulaceae)	Giant dodder	**Part:** Whole plant **Extract:** Methanol	IC_50_ <10 to 2.2 μg/mL	*In vitro*	Ojha et al. (2023)
*Caesalpinia bonducella* (Fabaceae)	Gray Nicker	**Part:** Root **Extract:** Dichloromethane	Norcaesalpin D IC_50_ 0.98–2.13 μg/mL	*In vitro*	Nondo et al. (2017)
• *Momordica charantia* (Cucurbitaceae) •*Diospyros monbuttensis* (Ebenaceae)	• Bitter melon •Yoruba ebony	**Part:** • Leaves • Leaves **Extract:** Alcohol	IC_50_ • 3.2 nM • 12.5 nM	*In vitro*	Olasehinde et al. (2014)
*Costus afer* (Costaceae)	Ginger lily	**Part:** Stem **Extract:** Aqueous	IC_50_ 10.3–15.05 μg/mL	*In vitro*	Jimoh et al. (2019)
*Rubia cordifolia* (Rubiaceae)	Indian madder	**Part:** Aerial **Extract:** (crude extraction) • Hexane • Methanol • Water	Dose: 50 μg/mL IC_50_ • 0.551 μg/mL • 1.23 μg/mL • 5.34 μg/mL	*In vitro*	Jeremiah et al. (2023)

### 8.3 *Helianthus annuus* L.

An Indonesian study investigated the *in vivo* and *in vitro* efficacy of *H. annuus* (Asteraceae). The ethanolic extracts from all parts of *H. annuus* were made separately. The root extract was more potent *in vitro* with IC_50_ values of 2.3 ± 1.4 μg/mL. In addition, leaf and flower extracts showed significant antimalarial activity with IC_50_ values of 4.3 ± 2.2 and 4.8 ± 0.0 μg/mL, respectively. *In vivo* studies in *P. berghei-*infected mice, treated with *H. annuus* root extract demonstrated the highest percentage inhibition, while leaf extract also showed significant inhibition (63.6 ± 8.0). Notably, the root extract *in vivo* curative assay showed a significant median effective (ED_50_) value of 10.6 ± 0.2 mg/kg. At 400 mg/kg dose, the group showed the highest prophylactic inhibition (79.2%) on day 3. Overall, the *H. annuus* extracts exhibited high activity against malaria (Ekasari et al., 2019; Table 4).

**TABLE 4:** Plants with anti-malaria properties.

Scientific name and family	Common name	Part used and extract	Dosing	Citation
*Artemisia annua* (Asteraceae)	Sweet wormwood	**Part:** Leaves **Extract:** Hydroalcohol	IC_50_ *In vitro*: 3.27 ± 1.42 nM *In vivo*: 20 mg/kg	Zime-Diawara et al. (2015)
*Helianthus annuus* (Asteraceae)	Common sunflower	**Part:** Whole plant **Extract:** Ethanol	IC_50_ *In vitro*: **Roots:** 2.3 ± 1.4 μg/mL **Leaf:** 4.3 ± 2.2 μg/mL **Flower:** 4.8 ± 0.0 μg/mL *In vivo*: 400 mg/kg ED50: 10.6 ± 0.2 mg/kg	Ekasari et al. (2019)
*Hypoestes forskaleii* (Acanthaceae)	White ribbon bush	**Part:** Leaves **Extract:** Crude, n-butanol	*In vivo*: 600 mg/kg	Misganaw etal. (2020)
*Angelica keiskei* (Apiaceae)	Tomorrow’s leaf	**Part:** Root	IC_50_ *In vitro*: 16.09 μg/mL	Wardani et al. (2020)
*Quercus infectoria* (Fagaceae)	Manja-kani	**Part:** Galls **Extract:** Acetone, methanol	IC_50_ *In vitro*: **Acetone extract:** 5.85 ± 1.64 μg/mL **Methanol extract:** 10.31 ± 1.90 μg/mL	Zin et al. (2020)
*Kniphofia foliosa* (Asphodelaceae)	Lella	**Part:** Rhizome **Extract:** Hydroalcohol	*In vivo*: **Hydroalcohol extract:** 400 mg/kg **Knipholone (phytocompound):** 200 mg/kg	Alebachew et al. (2021)
• *Physalis angulata* (Solanaceae) •*Jatropha curcas* (Euphorbiaceae) •*Alstonia spectabilis* (Apocynasceae)	• Cutleaf groundcherry •Physic nut •Bitterbark	**Part:** • Whole plant • Stem bark • Stem bark **Extract:** Ethanol	IC_50_ *In vitro*: • *P. angulate* 0.22 μg/mL • *J. curcas* 0.22 μg/mL • *A. spectabilis* 1.23 μg/mL	Maximus et al. (2021)
*Allium paradoxum* (Amaryllidaceae)	Few-flowered garlic	**Part:** Whole plant **Extract:** Hydroalcohol	*In vitro*: 80 μg/mL *In vivo*: 400 mg/kg	Elmi et al. (2021)

### 8.4 *Cyperus articulates* L.

A study focusing on the volatile oil obtained from rhizomes of the *C. articulates* (Cyperaceae) plant, commonly found in the Amazon region was carried out in Brazil. In this study, *P. falciparum* W2 (chloroquine-resistant) and 3D7 (chloroquine-sensitive) strains in erythrocytes were exposed to *C. articulates* volatile oils at different concentrations. The oil showed high activity against the two *P. falciparum* strains, with IC_50_ = 1.21 μg/mL for W2, and 2.30 μg/mL for 3D7. Furthermore, *in vivo,* antimalarial activity was tested in *P. berghei-*infected BALB/c mice. Treatment with *C. articulates* was a success in all 18 ACT-resistant cases (Daddy et al., 2017; Table 3).

The discovery of artemisinin has played a critical role in treating malaria, as it is not only active against various plasmodial forms, resistant to older drugs like chloroquine but also has fewer side effects. Its combination with other medications, such as lumefantrine, is crucial in combatting severe malarial infection (Marwa et al., 2022). With the development of different commercial forms of artemisinin-based drugs, there has been some improvement in the control over malaria endemics in the African and Mediterranean regions (Marwa et al., 2022). *C. articulates* volatile oil also significantly reduced parasitemia and anemia in animals treated with 100 and 200 mg/kg doses (Silva et al., 2019).

### 8.5 *Hypoestes forskaleii* (Vahl) R

*Hypoestes forskalei* (Acanthaceae) holds an eminent place in ethnopharmacology. In addition to its use as an anti-malaria, it is used as a cytotoxic, antimicrobial, larvicidal, antioxidant, antipyretic, antileismanial, and antitrypanosomal agent. To test *H. forskalei* anti-malaria effects, its leaf extracts were studied in both *in vitro* and *in vivo* settings. Notably, all the test doses of the crude extract and the fractions significantly reduced parasitemia and prolonged mean survival time (*p* < 0.001) as compared to their negative control groups. At 600 mg/kg dose of the crude extract during the 4-day suppressive test, maximum parasitemia suppression effect was observed (56%). At the same dose, the n-butanol, chloroform, and aqueous *H. forskalei* fractions revealed a percentage suppression of about 50, 38, and 19, respectively. Moreover, the n-butanol fraction, at a dose of 600 mg/kg, exhibited a significant curative effect (*p* < 0.001) in Rane’s test with a suppression of about 49% (Misganaw, Amare, and Mengistu, 2020; Table 4).

### 8.6 *Angelica keiskei (*Miq.) Koidz.

Chalcones is a renowned antimalarial phytocompound. *A. keiskei* (Apiaceae) is one of the plants that are rich in chalcone. A study aiming to determine the antimalarial activity of *A. keiskei* root extract by *in vitro* assay used the *P. falciparum* strain 3D7. The root extract, with an IC_50_ value of 16.091 μg/mL, exhibited an inhibitory effect as compared to chloroquine as a positive control with an IC_50_ value of 0.007 μg/mL. The study concluded that *A. keiskei* root extracts could be categorized as one of the antimalarial agents (Wardani, Wahid, and Rosa, 2020; Table 4).

### 8.7 *Quercus infectoria* G.Olivier

Similarly, a study conducted in Malaysia evaluated the anti-malaria potential of *Quercus infectoria* (Fagaceae). For this purpose, acetone, ethanol, methanol, and aqueous extracts were made from the *Q. infectoria* galls. Among these extracts, the acetone extract showed the highest antimalarial effects (IC_50_ = 5.85 ± 1.64 μg/mL), followed by the methanol extract (IC_50_ = 10.31 ± 1.90 μg/mL) against chloroquine-sensitive *P. falciparum*. Moreover, the study established the safety of these extracts, both acetone and methanol extracts were found to be non-toxic to the normal cell lines and statistically significant to artemisinin (*p* < 0.05) (Zin et al., 2020; Table 4).

### 8.8 *Pogostemon cablin* (Blanco) Benth

Another study conducted in Thailand found that the ethanol extract of *Pogostemon cablin* (Lamiaceae) carries the potential to become a more effective and safer anti-malarial agent. The efficacy and safety of the extract was tested by both *in vitro* and *in vivo* methods. *P. cablin* extract showed a significant IC_50_ of 24.49 ± 0.01 μg/mL against chloroquine-resistant *P. falciparum* K1. In comparison, the cytotoxic analyses revealed a nontoxic effect of the extract on Vero cells at a concentration of 80 μg/mL. In *P. berghei*-infected ICR mice, the ethanolic extract showed no toxic effect on mice at a dose of 2,000 mg/kg body weight. Notably, treatment with *P. cablin* extracts significantly suppressed parasitemia in mice by 38.41%, 45.12%, and 89.00% at doses of 200, 400, and 600 mg/kg body weight, respectively (Phuwajaroanpong et al., 2020; Table 3).

### 8.9 *Kniphofia foliosa* Hochst.

*K. foliosa* (Asphodelaceae) is indigenous to the Ethiopian highlands. Traditionally, its rhizomes are used for abdominal cramps, wound healing, as well as malaria management. To empirically establish *K. foliosa* as an anti-malaria agent, an investigation was carried out. In this study, two compounds (knipholone and dianellin) were isolated from the 80% methanolic extract of *K. foliosa* rhizomes. Upon evaluation by using Peters’ 4-day suppressive test against *P. berghei* in mice, the hydroalcoholic extract (400 mg/kg) and knipholone (200 mg/kg) demonstrated the highest activity with chemo suppression values of 61.52% and 60.16%, respectively. The dose-response plot analysis revealed the ED_50_ doses of knipholone and dianellin were 81.25 and 92.31 mg/kg, respectively. A molecular docking study revealed that knipholone had a strong binding affinity to the *P. falciparum* l-lactate dehydrogenase target (Alebachew et al., 2021; Table 4).

### 8.10 *Physalis angulata* L., *Jatropha curcas* L., *Alstonia spectabilis* R.Br.

To empirically understand the malaria treatment practices and evaluate the anti-plasmodial activity and phytochemicals of several plants used by the Tetun ethnic people in West Timor Indonesia, an experimental study was designed. In this study, ethanolic extracts from the whole *P. angulate* plant (family: Solanaceae), stem barks of *A. spectabilis* (family: Apocynaceae), and *Jatropha curcas* (family: Euphorbiaceae) were tested among others. Among the 11 plants studied in this investigation, *P. angulata, J. curcas,* and *A. spectabilis* extracts showed strong anti-plasmodial activity against the *P. falciparum* 3D7 strain *in vitro*, with IC_50_ values of 0.22, 0.22, and 1.23 μg/mL, respectively (Maximus et al., 2021) (Table 4).

### 8.11 *Allium paradoxum* (M.Bieb.) G.Don

A study aimed to examine the inhibitory effects of *A. paradoxum* (Amaryllidaceae) on *P. falciparum* and *P. berghei*. The highest efficacy of *A. paradoxum* extract was observed at 80 μg/mL in *P. falciparum* culture, resulting in 60.43% growth inhibition compared to control groups. The significantly highest parasite growth inhibition with 88.71% was seen in the mice infected with *P. berghei* when administered with 400 mg/kg extract compared to control groups. However, no significant changes in the liver and kidney cells were observed between the experimental and control groups (Elmi et al., 2021; Table 4).

### 8.12 *Toddalia asiatica* (L.) Lam., *Rhamnus prinoides* L'Hér., and *Vernonia lasiopus* O. Hoffm.

Extracts from various plant species were collected, authenticated, and processed using hot water extraction to mimic traditional methods. The activities of these extracts were tested against a chloroquine-resistant strain of *P. berghei* NK65 in mice. At the dose of 500 mg/kg, twice a day for 4 days, aqueous extract of *V. lasiopus* (root bark) and *R. prinoides* (leaves) showed remarkable suppression of parasitemia on day 4 post-infection (p.i.) with values of 51% and 54%, respectively. Moreover, the group treated with *Toddalia asiatica* (root bark) showed 100% survival up to day 15 p.i. Other extracts, including *R. prinoides* (leaves and root bark), showed prolonged survival with 40% of mice surviving beyond day 9 p.i. (Muregi et al., 2007; Table 3).

### 8.13 *Maytenus senegalensis* (Lam.) Exell, and *Rhamnus staddo* A.Rich.

In the same study by (Muregi et al., 2007), some plant extracts performed well when given in combination with chloroquine, particularly, extracts of *M. senegalensis, R. staddo, T. asiatica,* and *V. lasiopus,* in combination with chloroquine at a dose of 20 mg/kg, once a day for 2 days, and plant aqueous extracts at a dose of 500 mg/kg, twice a day for 4 days exhibited a remarkable suppression of parasitemia ranging from 51% to 66%. Survival analysis on day 14 p.i. showed that chloroquine in combination with certain plant extracts resulted in 40%–60% survival rates, with some mice surviving beyond day 14 p.i. Notably, combinations with *R. prinoides* (root bark) and *V. lasiopus* (root bark) showed 60% survival on day 14 p.i., with the last mice surviving beyond day 24 and day 30 p.i., respectively (Muregi et al., 2007; Table 3).

### 8.14 *Citrullus colocynthis* (L.) Schrad., *Physalis alkekengi* L., and *Solanum nigrum* L.

Drawing inspiration from Iranian traditional medicine, a study tested methanolic extracts from these plants against multi-drug resistant (K1) strains of *P. falciparum*. For the resistant strain, the study found that methanolic extracts of *C. colocynthis* exhibited promising *in vitro* anti-plasmodial activity, with an IC_50_ value of 6.9 μg/mL. Similarly, *Solanum nigrum* and *P. alkekengi* also showed activity against the resistant strain, with IC_50_ values of 18.67 and 13.08 μg/mL, respectively (Haddad et al., 2017; Table 3).

### 8.15 *Glycyrrhiza glabra* L.

In an *in vivo* investigation, the root extract of *Glycyrrhiza glabra* (Fabaceae) demonstrated anti-plasmodial potential. This activity was attributed to its phytomolecule: Isoliquiritigenin. Moreover, *in-vivo* antimalarial efficacy was evaluated through a 4-day suppression test in a mouse model. The phytocompound*, in vitro*, exhibited moderate anti-plasmodial activity against the multidrug-resistant strain (K1) of *P. falciparum*. Notably, the ethyl acetate extracts performed moderately against the resistant strain of *P. falciparum* (K1), with an IC_50_ value of 7.2 μg/mL. *In vivo*, mice infected with a chloroquine-resistant strain of *P. yoelli* nigeriensis were administered with the ethyl acetate extract at a dose of 500 mg/kg, which showed the highest inhibition of *P. yoelii* nigeriensis growth (82.73%), followed by doses of 250 mg/kg (64.40%) and 100 mg/kg (46.09%) compared to the control group. Also, the ethyl acetate extracts increased the mean survival time of the mice to 18.2 days, 13.5 days, and 16.5 days, respectively (Kumar et al., 2024; Table 3).

Interestingly, isoliquiritigenin was found to synergize with chloroquine and artemisinin against the multidrug-resistant strain (K1) of *P. falciparum*. The IC_50_ of chloroquine in the presence of isoliquiritigenin was found to drop from 0.132 to 0.013 μg/mL (10-fold). Importantly, the IC_50_ of isoliquiritigenin itself was found to drop from 5.2 to 1.90 μg/mL (up to 2.73-fold) in the presence of chloroquine. Similarly, with the isoliquiritigenin-artemisinin combination, it was found that the IC_50_ of artemisinin dropped from 3.9 to 1.75 ng/mL (2.22 times reduction). However, since all values fell within the range of >01 and ≤2, it indicated that the combination had an additive effect (Kumar et al., 2024; Table 3).

### 8.16 *Cuscuta reflexa* Roxb.

*C. reflexa* (Family: Convolvulaceae) has traditionally been used by the indigenous people of Odisha, India for the treatment of malaria. An *in vitro* evaluation was carried out to provide an empirical basis for this practice. *In vitro*, fractions of methanol extract from the shed-dried whole plant of *C. reflexa* exhibited promising effects (IC_50_ ranging from < 10.0 to 2.2 μg/mL) against *P. falciparum* R539T (artemisinin resistance strain) and RKL (chloroquine resistance strain) strains. Notably, no *in vitro* cytotoxicity was recorded. Moreover*, C. reflexa* fraction showed high *in vivo* parasite suppression, with a mean survival time similar to that of artesunate (19.3 vs 20.6 days) at a dose of 20 mg/kg in *P. berghei* ANKA-infected male Swiss-albino mice (Ojha et al., 2023; Table 3).

### 8.17 *Caesalpinia bonducella* (L.) Fleming

Another study explored the anti-malaria qualities of norcaesalpin D obtained from dichloromethane root extract of *C. bonducella* (Fabaceae). Additionally, crude extracts, fractions, and isolated compounds were evaluated for anti-plasmodial activity against chloroquine-resistant *P. falciparum* (Dd2, K1) and artemisinin-resistant *P. falciparum* strains. The results indicated the fractions from *C. bonducella* roots were found to be highly effective against K1, Dd2, and artemisinin-resistant parasites. Moreover, norcaesalpin D from *C. bonducella* root extract was active with IC_50_ of 0.98, 1.85, and 2.13 μg/mL against 3D7, Dd2, and IPC 4912-Mondolkiri parasites, respectively (Nondo et al., 2017; Table 3).

### 8.18 *Momordica charantia* L., and *Diospyros monbuttensis* Gürke

A Nigerian study tested the sensitivity of resistant *P. falciparum* species to plant extracts of *M. charantia, D. monbuttensis*, and *M. lucida* by using an *in vitro* micro-test (Mark III), based on assessing the inhibition of schizont maturation. Among the isolated parasites, all were sensitive to quinine, mefloquine, and artesunate; however, 51% of the isolates were resistant to chloroquine, 13% to amodiaquine, and 5% to sulphadoxine/pyrimethamine. The study found that the highest activity was obtained with an extract of *D. monbuttensis* with an IC_50_ of 3.2nM, while *D. monbuttensis* produced inhibitory activity with IC_50_ = 12.5 nM. However, the least satisfactory activity was obtained from *M. lucida* extract (IC_50_ = 25 nM) (Olasehinde et al., 2014; Table 3).

### 8.19 *Costus afer* Ker Gawl.

*C. afer* (Costaceae) was investigated for anti-malaria potentials *in vitro* activity by using its methanol stem extract and its residual aqueous fraction against chloroquine-resistant, and artemether-resistant *P. falciparum* strains. The extracts demonstrated significant and dose-dependent inhibitions of schizont growth in the resistant *Plasmodium* strains with IC_50_ values of 11.27 and 15.05, and 10.30 and 11.23 μg/mL against, chloroquine-resistant and artemether-resistant strains, respectively (Jimoh et al., 2019; Table 3).

### 8.20 *Rubia cordifolia* L.

*R. cordifolia* is part of Kenyan traditional medicine for malaria treatment; however, there is limited scientific evidence supporting this practice. To begin addressing this gap, a study aimed to assess the plant-extract efficacy, taken from the aerial part of *R. cordifolia*, against chloroquine-resistant (W2) *P. falciparum* strains. Major findings of the study included a significant inhibition of parasite growth, in a dose-dependent manner. At 50 μg/mL, inhibition was (84.07%, 77.94%, and 66.08%) for hexane, methanol, and water extracts, respectively, against the resistant strain. Hexane extract showed an IC_50_ 0.551 μg/mL, while methanol extract gave encouraging results with an IC_50_ 1.231 μg/mL. However, the aqueous extract showed moderate activity (IC_50_ = 5.348 μg/mL) (Jeremiah et al., 2023; Table 3).

## 9 Medicinal plants used for malaria treatment in Pakistan

The Himalayan region of Pakistan is renowned for its diverse herbal remedies the local communities use to treat various health problems. A study on these plants found that Asteraceae (11.9%) was the most cited vegetal family against malaria. Other plant families being used included Lamiaceae (5.9%), Solanaceae, Verbenaceae (4.7%), and Violaceae (3.5%). A study conducted by (Shah and Rahim, 2017), in the Soon Valley of Pakistan, indicated the use of more than fifty varieties for malaria treatment in the region. The researchers identified that anti-malarial properties of seven plant species were reported for the first time. These plant species included *Withania coagulans* (Stocks), *Fagonia cretica* L. (Zygophyllaceae), *Carthamus oxyacanthus* M.Bieb. (Asteraceae), *Ehretia obtusifolia* Hochst. ex A.DC. (Boraginaceae), *Helianthus annuus* L. (Asteraceae), *Olea ferruginea* (Aiton) Steud. (Oleaceae), and *Vitex trifolia* L. (Lamiaceae).

Another study focused on three local plants for their anti-malarial activity. The plant species analyzed included *P. kurroa*, *C. bonducella*, and *A. absinthium*. The investigators used cold alcohol, hot alcohol, and aqueous preparations of extracts obtained from the plants to ascertain their antimalarial activity. The study found that *P. kurroa* was more effective than the other two plants against malaria (Irshad, Mannan, and Mirza, 2011).

## 10 Discussion

One of the strengths of this review is its emphasis on the use of natural products for malaria treatment. Natural remedies for treating malaria have several advantages over mainstream drugs. First, natural products offer a vast array of chemical structures with diverse pharmacological properties (Dias, Urban, and Roessner, 2012). This chemical diversity provides a rich source of compounds with potential anti-malarial activity, allowing for the discovery of novel drugs or drug leads. This chemical diversity also helps in delaying the emergence of drug resistance. The prevention or at least delay of drug resistance may also be helped by the fact that many natural products exhibit activity against multiple stages of the malaria parasite’s life cycle, including the blood stages (schizonts), liver stages (hypnozoites), and transmission stages (gametocytes) (Roberts et al., 2017). This multifactorial action may help prevent the development of drug resistance and improve treatment outcomes. Natural products often contain multiple bioactive compounds that can act synergistically to enhance their anti-malarial activity. This synergism may result in greater efficacy and lower risk of drug resistance compared to single-compound therapies. Most natural products exhibit anti-malarial activity with minimal toxicity to human cells, making them potentially safer alternatives to synthetic drugs (Amelo, Nagpal, and Makonnen, 2014). This is particularly important in malaria-endemic regions where access to healthcare and monitoring for adverse drug reactions may be limited. This review not only focuses on the use of natural products for treating malaria but emphasizes that combining treatment with a hybrid approach of vector control methods with rigorous hygiene measures can be an effective strategy for combating malaria. Especially, the distribution of insecticide-treated bed nets and indoor residual spraying with long-lasting insecticides can significantly reduce mosquito populations and prevent malaria transmission indoors (Hellewell et al., 2021). Targeted application of larvicides to mosquito breeding sites, such as stagnant water bodies, can prevent the development of mosquito larvae and reduce adult mosquito populations (Lardeux et al., 2002). Implementing an integrated approach that combines multiple vector control methods, tailored to local epidemiological and ecological conditions, can enhance the effectiveness and sustainability of malaria control efforts. Establishing robust surveillance systems to monitor malaria transmission dynamics, vector populations, and insecticide resistance can inform evidence-based decision-making and guide the adaptation of control strategies over time.

## 11 Limitations of the herbal treatments

An important limitation of herbal medicine is the variation in the potency and effectiveness that may occur due to several factors, including geographic variations in malaria parasite strains, drug resistance patterns, and differences in local healthcare infrastructure and practices. For example*, P. falciparum* is the most prevalent and deadliest of the malaria parasite species, and resistance to anti-malarial drugs, such as chloroquine and sulfadoxine-pyrimethamine, has been widely reported in many regions, particularly in sub-Saharan Africa (Crider et al., 2022). National and international treatment guidelines for malaria may vary based on epidemiological data, drug resistance patterns, and healthcare resources. Treatment recommendations may be tailored to specific regions or populations to optimize treatment outcomes and minimize the risk of drug resistance. For example, ACTs are recommended as first-line treatment for uncomplicated *P. falciparum* malaria in most endemic regions, but the specific ACT regimen may vary based on local drug resistance patterns (Saito et al., 2023).

Another limitation of herbal treatments is the lack of standardized dosing for malaria which can have several implications, including variability in treatment efficacy, safety concerns, and challenges in comparing research findings across studies. Natural product treatments often contain complex mixtures of bioactive compounds, which may vary in concentration and composition depending on factors such as plant species, growing conditions, harvesting methods, and processing techniques (Santos-Zea et al., 2016). This variability makes it difficult to establish standardized dosing regimens across different preparations of the same natural product. Many natural products lack comprehensive pharmacokinetic data, including information on absorption, distribution, metabolism, and excretion (ADME) in humans. Without this data, it is challenging to determine optimal dosing regimens that achieve therapeutic levels of active ingredients while minimizing the risk of toxicity or suboptimal efficacy. Natural products may contain toxic compounds or interact with other medications, leading to adverse effects or drug interactions (Fugh-Berman, 2000). Without standardized dosing and rigorous safety evaluation, there is a risk of overdosing, under-dosing, or unintended adverse reactions, particularly in vulnerable populations such as children, pregnant women, or individuals with underlying health conditions.

The potential interactions of natural products such as for malaria with other medications are an important consideration, as they can impact treatment efficacy, safety, and patient outcomes, thus limiting their use (Fugh-Berman, 2000). While natural products are often perceived as safe, they contain bioactive compounds that may interact with conventional medications that may alter their pharmacokinetics or pharmacodynamics. Natural products may affect the ADME of other medications, leading to alterations in their blood levels and therapeutic effects. For example, certain natural products may inhibit or induce drug-metabolizing enzymes in the liver (e.g., cytochrome P450 enzymes), influencing the metabolism of co-administered medications and potentially leading to drug toxicity or treatment failure (Yang, Wang, and Zeng, 2002; Parvez and Rishi, 2019). Furthermore, natural products may have additive, synergistic, or antagonistic effects when combined with other medications, altering their pharmacological actions. For instance, some natural products may enhance the anticoagulant effects of blood-thinning medications or potentiate the sedative effects of central nervous system depressants. Similarly, St. John’s wort (*Hypericum perforatum* L.), a herbal remedy sometimes used for its antidepressant effects, could induce drug metabolizing enzymes and reduce the efficacy of medications, including certain anti-malarial drugs, oral contraceptives, and immunosuppressants (Yang, Wang, and Zeng, 2002).

## 12 Conclusion

Malaria remains a formidable global health challenge, causing significant morbidity and mortality, particularly in impoverished regions. Despite numerous diagnostic and medicinal breakthroughs, the *Plasmodium* resistance development to anti-malaria drugs will undo most of this progress. This threat has become an impetus behind the race for novel, effective, and affordable drugs and vaccines. Natural products derived from plants like *A. annua, C. sanguinolenta, H. impetiginosus, P. kurroa,* and *A. hexapetalus* are among the noteworthy sources. Interestingly, *A. annua* is the precursor of artemisinin, one of the most significant drugs considered a mainstay of treatment against malaria. Moreover, plants like *V. lasiopus*, *R. prinoides*, *T. asiatica, C. colocynthis,* and phytocompounds like isoliquiritigenin, and norcaesalpin D should be further studied for effects against drug-resistant *Plasmodium* species. However, while herbal medicines have advantages like cost-effectiveness and fewer side effects, they also have limitations. These limitations include variations in potency, lack of standardized dosing, and potential interactions with other medications. Therefore, either modern or herbal medicines should be used after conducting a cost-benefit analysis associated with the treatment plan. Only a balanced approach can have an effectual impact. In addition, an emphasis on early detection, accurate diagnosis, and integrated efforts for disease prevention is also crucial. Additionally, implementing stringent hygiene measures and promoting community awareness will also contribute to reducing the burden of malaria in endemic areas.
